# Evaluation of the Optic Disc and Macula in Healthy Children Using Optical Coherence Tomography Angiography

**DOI:** 10.4274/tjo.galenos.2020.85282

**Published:** 2020-08-26

**Authors:** Erel İçel, Hayati Yılmaz, Turgay Uçak, Nurdan Gamze Taşlı, Adem Uğurlu, Yücel Karakurt

**Affiliations:** 1 Erzincan Binali Yıldırım University Faculty of Medicine, Department of Ophthalmology, Erzincan, Turkey

**Keywords:** Retina, optic coherence tomography, vessel density, foveal avascular zone, Nidek RS-3000 Advance

## Abstract

**Objectives:**

To perform the measurements of the optic disc and macula in healthy children using optical coherence tomography angiography (OCTA) in order to determine the normative data values and compare these by age, gender, spherical equivalent (SE), and axial length (AL).

**Materials and Methods:**

A total of 146 eyes belonging to 146 healthy children (74 girls, 72 boys) aged 6 to 16 years were included in this prospective study. Refraction and biometry measurements were performed. Retinal nerve fiber layer thickness (RNFLT), central macular volume, and central macular thickness (CMT) were measured by optical coherence tomography (OCT) after dilatation. Using the OCTA device, foveal avascular zone (FAZ) area, vascular density (VD) of the superficial capillary plexus (SCP) and deep capillary plexus (DCP) of the macula, and the VD of the radial peripapillary capillary plexus (RPCP) of the optic disc were recorded.

**Results:**

The mean age of the study group was 11.27±3 years, the mean AL was 23.39±1.18 mm and the mean SE was -1.31±1.61 diopters. The mean FAZ area was 0.3±0.09 mm^2^, the mean SCP-VD was 43.88±3.4%, the mean DCP-VD was 39.6±3.55%, and the mean RPCP-VD was 52.47±3.42%. When the relationship between the OCTA measurements and the SE and AL values were analyzed, there was no statistical significance (p>0.05). When age and OCTA measurements were compared, only DCP-VD values were found to significantly decrease with increasing age (p=0.015). There was no significant difference in OCTA measurements based on gender (p>0.05). Similarly, no statistical age-based differences were observed in RNFLT, CMV and CMT values (p>0.05).

**Conclusion:**

With its short procedure time and no dye requirement, OCTA can be safely used in the evaluation of the optic disc and macular perfusion in children. Determination of normative values in children will be useful in detecting pathologic changes in tissue in patients with retinal diseases.

## Introduction

Optical coherence tomography (OCT) is a widely used, noninvasive imaging method that offers high-resolution cross-sectional scanning of ocular tissues. However, OCT cannot clearly identify blood vessels due to the scattering of light caused by the movement of red blood cells.^1^ This scattering creates a shadow effect behind or under the blood vessels, causing image loss in the deep layers of the region.^2^ In addition, it does not show leakage or perfusion disorders in the retina and the choroid. To observe these changes, methods that involve the use of dye during scanning, such as fluorescein angiography (FA) and indocyanine green angiography are used. However, these methods have the disadvantages of producing two-dimensional, low-resolution images and causing adverse effects such as nausea, vomiting, and allergic reactions.^2,3^

With the increased sensitivity and speed of OCT systems, it is now possible to visualize blood vessels through repeated scans. OCT angiography (OCTA), obtained from the development of spectral domain OCT (SD-OCT), provides detailed three-dimensional data on the microvascular structure of the retina and the choroid.^4^ Using sequential OCT B-scans offered by this technology, vascular networks can be visualized by processing the contrast of erythrocyte movements in the vessels in a specific retinal area.^5^ In recent research, this method has been reported to provide easy diagnosis and follow-up in ocular diseases, including diabetic retinopathy and glaucoma.^6,7^

Glaucoma and retinal diseases may cause vision loss in childhood. Ophthalmoscopy used in the diagnosis is a subjective method for evaluating retinal damage.^8^ In pediatric cases, there may be problems related to diagnosis and follow-up due to patient noncompliance with procedures requiring a long exposure time, such as FA and the visual field test. In contrast, the OCTA device can easily be used in children to facilitate diagnosis and follow-up.

To the best of our knowledge, the OCTA findings of healthy pediatric cases using the AngioScan software of the Nidek RS-3000 Advance OCT system have not been previously reported in the literature. Therefore, in this study, we aimed to evaluate the optic disc and macula using the OCTA device with children in order to determine the normative values in this group. In addition, we analyzed the relationships between these values and age, gender, spherical equivalent (SE), and axial length (AL). In this way, we planned to obtain reference values for pediatric patients with retinal diseases such as diabetic retinopathy, or those who may have optic disc damage due to glaucoma.

## Materials and Methods

This prospective observational cross-sectional study was performed in the ophthalmology department of Erzincan Binali Yıldırım University, Faculty of Medicine, Mengucek Gazi Training and Research Hospital. The study protocol was approved by the local ethics committee (Date: 10.09.2019 No: 33216249-604.01.02-E.43258). Written informed consent was obtained from the parents or legal guardians of all participants.

### Study Population

Healthy children aged 6 to 16 years who presented to the ophthalmology clinic for routine examination were included in the study. The inclusion criteria were as follows: children who were born at full term (≥37 weeks gestational age) with normal birth weight (≥2.500 g), refractive error ≤±4 diopters (D), best-corrected visual acuity ≥20/20, intraocular pressure level of both eyes ≤21 mmHg, and no optic disc or macular pathology on mydriatic fundus examination. The exclusion criteria were specified as the presence of any systemic disease; the presence of any ocular pathology such as strabismus, amblyopia, and glaucoma; and a family history of optic nerve or retinal pathologies. Patients who did not meet these criteria, those who did not comply with the OCT and/or OCTA procedure, and those whose parents did not provide written consent were excluded from the study.

The demographic data of all cases were recorded, and detailed eye examinations were performed. The AL measurements of the participants were performed by biometry (ALSCAN, Nidek Co. Ltd., Aichi, Japan), and 1% cyclopentolate drops were applied to both eyes 3 times at 5-minute intervals. After 30 minutes, autorefraction measurements were performed using an autorefraction device (Tonoref III, Nidek Co. Ltd., Aichi, Japan). The measurements were repeated 3 times in both eyes. In addition, SE values (spherical error + 50% of cylindrical error) were calculated from the average refractive error values for each eye. Intraocular pressure levels (Tonoref III, Nidek Co. Ltd., Aichi, Japan) were recorded and slit-lamp biomicroscopic examination was performed. Macular and peripapillary thicknesses were measured using an SD-OCT device (Nidek Co. Ltd., Aichi, Japan). Foveal avascular zone (FAZ) area, vessel density (VD) of the superficial capillary plexus (SCP) and deep capillary plexus (DCP) of the macula, and the VD of the radial peripapillary capillary plexus (RPCP) of the optic disc were quantified by OCTA (RS-3000 Advance, Nidek Co. Ltd., Gamagori, Japan).

### Scan Protocol

The Nidek RS-3000 Advance OCT system and updated AngioScan software were used to evaluate SD-OCT and OCTA images. The light source of this device has a wavelength of 880 nm and has optical resolution of 7 µm on the z axis, 20 µm on the x and y axes, and a speed of 53,000 A-scans per second. The fovea is focused on using an OCTA prototype internal fixation lamp, and 3x3 mm macula cubes, each consisting of 256 B-scans are generated. Recently, Nidek developed a new version of the AngioScan software. With this update, the macular and peripapillary VD can be automatically calculated, as well as FAZ area. Only the FAZ area in the DCP was calculated manually because this measurement cannot be performed automatically using this device.

SD-OCT and OCTA measurements were performed by an experienced clinician after pupil dilation. Scans with signal strength index <7/10 were repeated. The scans consisted of a 3×3 mm macular map centered on the fovea and a 2.4x4 mm disc map centered on the optic disc. The tracing HD plus function of the Nidek RS-3000 Advance system reduces motion and blink artifacts.

**Figure 1:**
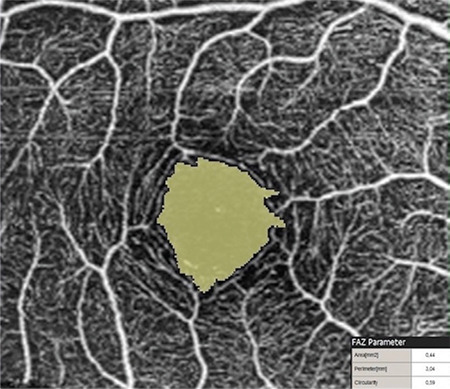
Optical coherence tomography angiography image showing FAZ measurement in the superficial capillary plexus layer FAZ: Foveal avascular zone

**Figure 3:**
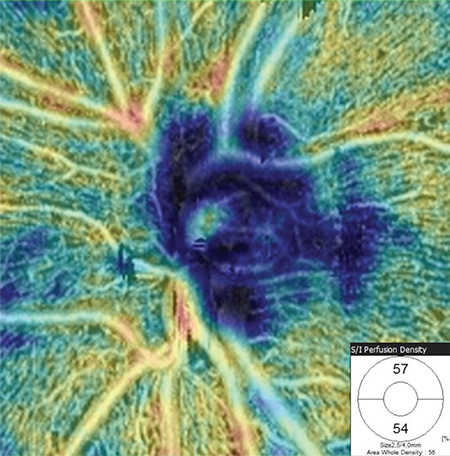
Peripapillary vessel density colored map at the level of the radial peripapillary capillary plexus (RPCP) of a healthy subject and vessel density distribution for the superior and inferior RPCP hemifields

FAZ area and VD in the SCP and DCP were measured in both eyes of all participants. For the superficial plexus, the FAZ was automatically calculated. An OCTA image showing FAZ measurement in the SCP layer is given in Figure 1. Capillary VD was measured in both the SCP and DCP of the macula for quantitative evaluation of the microvasculature. VD was calculated as the percentage of area occupied by flowing blood vessels in the selected region. OCTA images showing retinal VD measurements in the SCP and DCP are presented in Figure 2a and 2b. RPCP-VD in the peripapillary region was also recorded (Figure 3). Retinal nerve fiber layer thickness (RNFLT), central macular thickness (CMT), and central macular volume (CMV) were measured through SD-OCT analysis. Macular thickness was measured in the 9 sectors included in the macular chart of the Early Treatment Diabetic Retinopathy Study (ETDRS).

### Statistical Analysis

SPSS v. 21.0 was used for statistical evaluation. The Kolmogorov-Smirnov test was carried out to determine the distribution of the data. Normally distributed data were analyzed using Spearman’s correlation analysis, and Pearson correlation test was employed to evaluate the relationship between nonnormally distributed variables. Independent t-test was used for gender comparisons. The statistical significance level was accepted as p<0.05.

## Results

**Study group: **A total of 163 healthy children that presented to our clinic for routine examination during the study period met the inclusion criteria. Three children did not have parental consent and 14 children did not comply with the OCTA measurement process; thus, a total of 146 random eyes belonging to 146 children (74 [51%] girls and 72 [49%] boys) were included in the study. The mean age of the participants was 11.27±3 years.

**Measurement results: **The mean AL of the eyes was 23.39±1.18 mm and the mean SE was -1.31±1.61 D. The mean values of RNFLT, CMT, and CMV were 105.6±11.66 µm, 257.56±18.73 µm and 9.13±0.59 mm^3^, respectively, and there was no significant difference in these variables according to age (p=0.31, p=0.88, p=0.67, respectively).

The mean FAZ area was 0.3±0.09 mm^2^, the mean SCP-VD was 43.88±3.4%, and the mean DCP-VD was 39.6±3.55%. For the optic disc measurements, the mean RPCP-VD value was 52.47±3.42%. No significant difference was found in FAZ, SCP-VD, or RPCP-VD according to age, but DCP-VD decreased significantly with increasing age (p=0.52, p=0.32, p=0.96, and p=0.015, respectively). Neither SE nor AL showed a significant relationship with FAZ, SCP-VD, DCP-VD, and RPCP-VD (p>0.05).

When the relationship between FAZ area and the other parameters was examined, it was determined that SCP-VD, DCP-VD, RPCP-VD, CMT, and CMV significantly decreased as FAZ area increased (p=0.008, p=0.004, p=0.036, p<0.0001, and p=0.023, respectively).

SE, AL, RNFLT, CMT, CMV, FAZ, SCP-VD, DCP-VD, and RPCP-VD values did not differ significantly between boys and girls (p>0.05) (Table 1).

## Discussion

Recently, OCTA has become a widely used device due to its short procedure time, noninvasive nature, no requirement of dye use during scanning, and no adverse effects on the patient.^6^ Considering the long exposure time and the requirement of dye use in other imaging methods such as FA, OCTA presents a useful and easy way of detecting retinal and optic disc pathologies, especially in pediatric cases.^9^

Nidek has recently updated the AngioScan software, but there are limited normative data for the new version of the device in the literature.^4^ Since different techniques are used in different OCTA devices, it is not possible to standardize measurements.^10,11^ When we reviewed the literature, we observed that normative data for the OCTA screening parameters using RS-3000 Advance had not yet been published for pediatric cases.

In this study, the OCTA device was used to measure the FAZ in the SCP layer, VD in the SCP and DCP layers, and VD of the RPCP of the optic disc in 146 healthy children. This is the first study to evaluate OCTA data in healthy Turkish children.

Adult studies predominate in the literature related to OCTA. These studies have reported morphological changes in FAZ area and VD in retinal diseases such as diabetic retinopathy and retinal venous occlusion in adult samples.^12,13^

The data on OCTA findings in pediatric cases are limited.^14,15,16^ Zhang et al.^17^ evaluated healthy children with a mean age of 11.51±1.91 years and reported the mean FAZ area to be 0.290±0.109 mm^2^, which is smaller than the mean FAZ area we obtained from a similar age group in our study. This may be due to the differences between the two studies in terms of the sample size, ethnicity of the participants or measurement device. In the same study, it was observed that FAZ area was significantly larger in girls than in boys.^17^ In another study conducted with healthy and diabetic children, the FAZ was smaller in males than females in both the study and control groups.^18^ In contrast, in the current study, there was no significant difference between the sexes when FAZ values were compared (p=0.726). Similarly, Samara et al.^19^ found no significant difference in FAZ area between the female and male participants. Whether sex has an effect on FAZ can only be clearly determined in further studies with a larger population.

In studies conducted with adults, FAZ area varies between 0.25 mm^2^ and 0.35 mm^2^.^20,21^ Although different devices and measurement methods are used, FAZ values in healthy children can be considered similar to healthy adults. In our study, a negative correlation was found between FAZ area and SCP-VD and DCP-VD. In addition, as FAZ area increased, CMT decreased, which is consistent with previous studies.^22,23^

When the effect of age on OCTA measurements was examined, only DCP-VD significantly decreased with increasing age (p=0.015). Similar studies have shown that both SCP-VD and DCP-VD values are significantly associated with age.^24,25^ Like Bazvand et al.^26^, we observed that RPCP-VD was not affected by age or sex. In contrast to the study by Yu et al.^27^, we did not detect any relationship between RPCP-VD and RNFLT. These comparisons support the idea that the results of studies may differ due to the different measurement techniques of the devices used and the ethnicities of the participants.

### Study Limitations

Limitations of the study include the small number of participants, the exclusion of children aged under the age of 6 years due to noncompliance, the common ethnic origin of the sample, the automatic measurement of FAZ and VD values by the device software, and reduced objectivity in the comparison of the results with previous research due to the use of updated software of the device in the current study. Another limitation concerns the manual measurement of FAZ area in the deep layer. Despite these limitations, we believe the results of the present study are valuable.

## Conclusion

In conclusion, this study evaluated normative data in healthy Turkish children using the Nidek OCTA device with the recently updated AngioScan software to enable future comparisons with data from cases with pathological conditions. There is a need for future studies to compare the presented data with those obtained from other devices and evaluate the normative data for pediatric cases in a larger population.

**Statement:** This study was presented as a poster at the 53rd National Ophthalmology Congress held November 6-10, 2019 in Antalya, Turkey.

## Figures and Tables

**Table 1:**
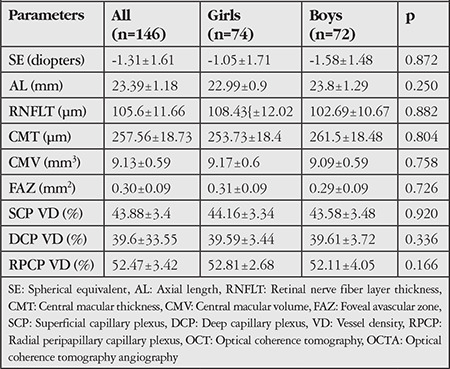
Comparison of clinical characteristics and OCT and OCTA measurements of the cases by gender

**Figure 2a:**
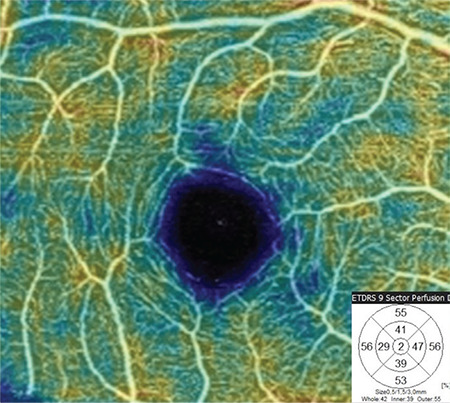
Colored vessel density map at the superficial capillary plexus level of a healthy subject and vessel density distribution according to the 9-sector Early Treatment Diabetic Retinopathy Study (ETDRS) chart

**Figure 2b:**
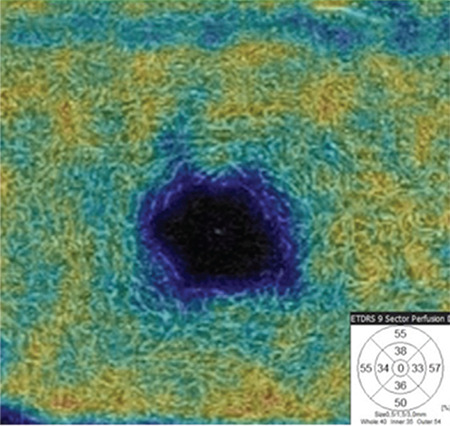
Colored vessel density map at the level of the deep capillary plexus of a healthy subject and vessel density distribution according to the 9-sector Early Treatment Diabetic Retinopathy Study (ETDRS) chart
